# Physical activity and carotid atherosclerosis risk reduction in population with high risk for cardiovascular diseases: a cross-sectional study

**DOI:** 10.1186/s12889-022-12582-6

**Published:** 2022-02-07

**Authors:** Lulu Chen, Yuan Bi, Jian Su, Lan Cui, Renqiang Han, Ran Tao, Jinyi Zhou, Ming Wu, Yu Qin

**Affiliations:** 1 Department of Non-communicable Chronic Disease Control, Jiangsu Provincial Center for Disease Control and Prevention, Nanjing, China; 2 Department of Epidemiology and Health Statistics, School of Public Health, Southeast University, Nanjing, China; 3 Shanghai Municipal Center for Health Promotion, Shanghai, China

**Keywords:** Physical activity, Carotid intima-media thickness, Sedentary leisure time, Carotid plaque, Age

## Abstract

**Background:**

Decreased physical activity had been reported to be a common causal and modifiable risk factor for major vascular events. However, the relationship of physical activity and sedentary leisure time with carotid atherosclerosis in population with high risk for cardiovascular diseases (CVDs) is still inconclusive. We aimed to evaluate the association of physical activity and sedentary leisure time with the risk of carotid atherosclerosis, and investigate any possible effect modifiers in population with high risk for CVDs.

**Methods:**

The study population was drawn from the China Patient-Centered Evaluative Assessment of Cardiac Events (PEACE) Million Persons Project-Jiangsu project, which is a population-based screening project that included permanent residents aged 35-75 years from 6 surveillance cities in Jiangsu Province. Linear regression models were used to evaluate the association of physical activity and sedentary leisure time with carotid intima-media thickness (CIMT). The risks of abnormal carotid artery and carotid plaque (CP) were estimated by odds ratios (ORs) and 95% confidence intervals (CIs) using logistic regression.

**Results:**

Overall, a total of 10,920 participants were enrolled in the final analysis. There was a significant inverse association of physical activity level with CIMT (per SD increase: β=-0.0103; 95%CI: -0.0154, -0.0053). The risk of abnormal carotid artery and CP decreased significantly with the increase of physical activity level (per SD increase: OR=0.908, 95%CI: 0.869-0.948; OR=0.900, 95%CI: 0.857-0.945, respectively). When physical activity level was categorized as quartiles, a significantly lower risk of abnormal carotid artery and CP was found in quartiles 2-4 with quartile 1 as reference (*P*<0.05 for all). Furthermore, the inverse association were stronger in participants with age ≥60 years (vs. <60 years, *P*_interaction_<0.001 for both). However, there were no significant association of sedentary leisure time with CIMT, abnormal carotid artery and CP.

**Conclusions:**

In population with high risk for CVDs, physical activity was inversely associated with CIMT, abnormal carotid artery and CP, particularly among the elders. Sedentary leisure time was not associated with them. These results suggested that physical activity is important for carotid vascular health, and perhaps especially in elder population.

**Supplementary Information:**

The online version contains supplementary material available at 10.1186/s12889-022-12582-6.

## Introduction

In recent years, cardiovascular diseases (CVDs) is the leading cause of disability and premature death worldwide, representing a severe public health problem [1]. By 2030, approximately 23.6 million people are predicted to die from cardiovascular diseases annually [2]. Atherosclerosis, as the main pathological process of most cardiovascular diseases, can reflect early vascular damage and predict future cardiovascular outcomes, and the early detection of atherosclerosis mainly focus on carotid artery [3, 4]. Carotid intima-media thickness (CIMT) and carotid plaque (CP) are usually used as markers of subclinical atherosclerosis which could reflect the occurrence of CVD independently of traditional CVD risk factors [5]. In 2020, the prevalence of carotid atherosclerosis (CAS) in people aged 30-79 years is estimated to be 48.7% globally, equivalent to 1882.46 million affected people and a percentage change of approximately 58% from 2000 [2]. At the same time, the number of people affected by CAS and CP will increase to 267.25 million and 199.83 million, respectively, by 2020 [6]. An upward trend of prevalence of CAS is very likely in the foreseen future, therefore, the huge and still growing prevalence of CAS and CP indicates that identifying modifiable risk factors for implementation of preventive measures is urgently needed.

Increasing physical activity level and reducing sedentary leisure time had been reported to be common causal and modifiable risk factors for major vascular events [7, 8]. This protective effect may be attributed to improvements in blood pressure, plasma lipoprotein profile, insulin sensitivity, and mood [9]. Several studies have investigated the association of physical activity level and sedentary leisure time with CIMT and CAS [10–14]. Most studies observed a relationship of higher physical activity and lower sedentary time with lower CIMT [10–12], and less CAS [10, 12]. However, two previous population-based studies didn’t show the relationship [13, 14]. Therefore, the relationship of physical activity level and sedentary leisure time with CAS remain unclear. Moreover, populationwide public health approaches alone will not have an immediate tangible impact on cardiovascular morbidity and will have only a modest absolute impact on the disease burden. By themselves they cannot help the millions of individuals with high risk for CVDs [15]. How to target individuals with high risk for CVDs, using cost-effective preventive approaches is urgently needed. Meanwhile, current guidelines for CVD prevention generally recommend a healthy lifestyle, particularly regular physical activity [15] which are mainly based on data from studies in general population. There is currently insufficient data from individuals with high risk for CVDs.

Therefore, we aimed to explore whether there is an association of physical activity level and sedentary leisure time with CAS assessed by CIMT, CP and abnormal carotid artery, and to examine the possible effect modifiers in population with high risk for cardiovascular diseases (CVDs) in China.

## Materials and methods

### Study design and participants

**Fig. 1:**
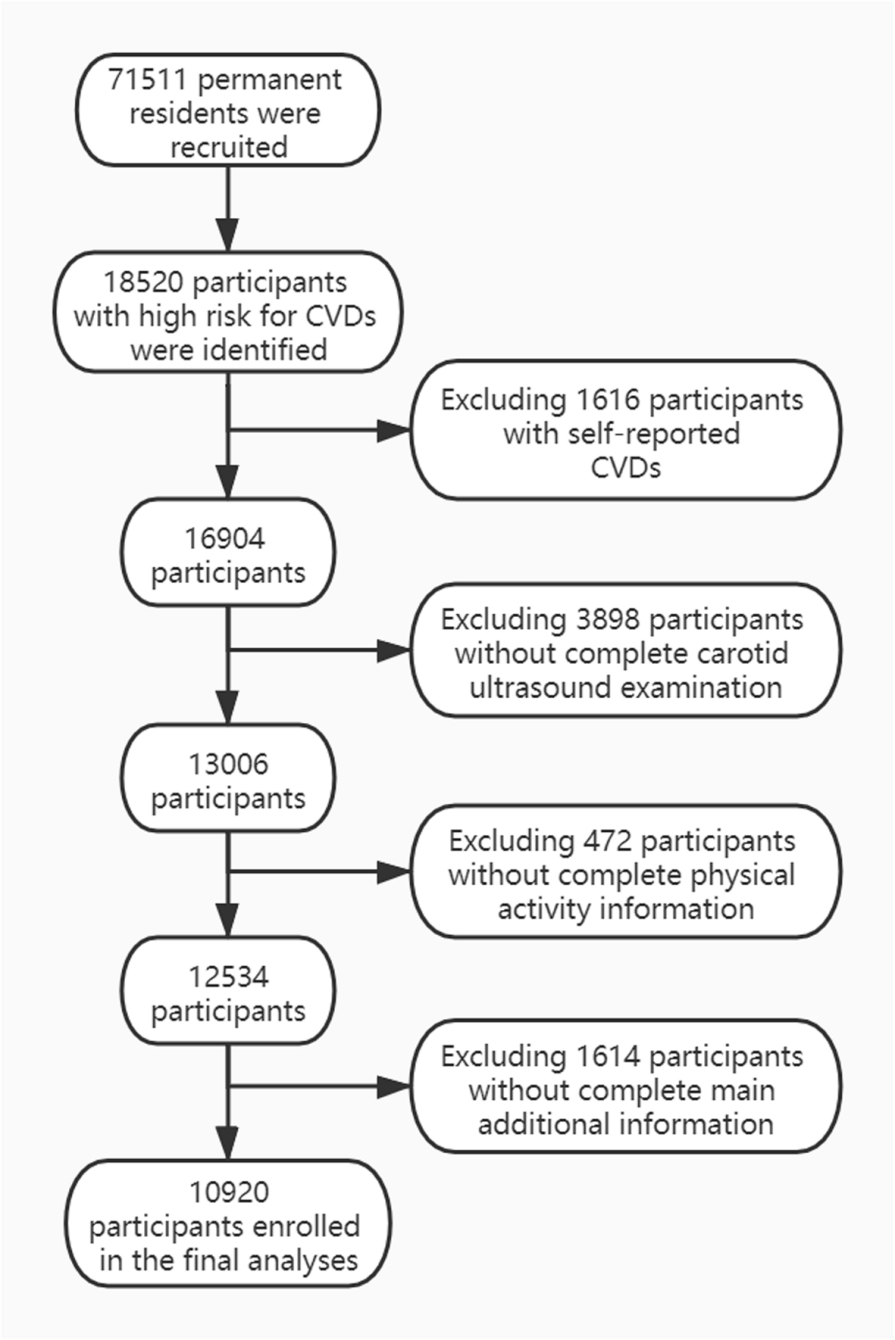
Flow chart of the participants enrolled in the final study

A government-funded public health program was conducted in 2015 in Jiangsu province, which is part of China PEACE (Patient-centered Evaluative Assessment of Cardiac Events) Million Persons Project [16], launched by China National Center for Cardiovascular Diseases (NCCD). The project is consisted of 3 stages: screening and recruitment, a short-term (3-month) and a long-term (12-month) follow-up, with the objective of identifying population with high risk of CVDs and counseling potential lifestyle changes. From 2015 to 2016, 71,511 permanent residents aged 35 to 75 from 6 surveillance sites (Changzhou, Huaian, Jiawang, Changshu, Haian, Donghai) in Jiangsu Province of China were recruited by convenience sampling. Participants with high risk for CVDs are defined as meeting one of the following criteria: (1) history of any disease or treatment of myocardial infarction (MI), ischemic or hemorrhagic stroke, percutaneous coronary intervention (PCI) or coronary artery bypass graft (CABG); (2) systolic blood pressure (SBP) ≥160 mmHg or diastolic blood pressure (DBP) ≥100 mmHg; (3) low-density lipoprotein cholesterol (LDL-C) ≥4.14 mmol/L or high-density lipoprotein cholesterol (HDL-C) <0.78 mmol/L; (4) a 10-year risk of CVD ≥20% [15]. Overall, 18,520 participants with high risk for CVDs were screened. Among them, 7600 participants with a history of CVD or with incomplete data were excluded. Finally, a total of 10,920 participants were enrolled in the analysis (Fig. 1). The study was approved by the Central Ethics Committee of the China National Center for Cardiovascular Disease, Beijing, China. All enrolled participants provided written informed consent.

### Data collection

During the initial screening stage, each participant received face-to-face questionnaire interview, physical examination and laboratory measurement.

The questionnaire included information on: (1) sex, age and other general demographic data; (2) smoking and drinking status; (3) education and annual household income; (4) history of diseases and treatment. Anthropometric measurements included height, weight and waist circumference (WC). Participants were required to wear light clothes, with no shoes and no headgear while being measured for height and weight. Body mass index (BMI) was calculated by dividing weight (kg) by height (m^2^). Seated blood pressure was measured twice on the right upper arm after 5 min of rest with an automatic digital sphygmomanometer (Omron HBP-1300; Omron Corporation, Kyoto, Japan). If the difference between the two SBP readings was greater than 10 mmHg, a third measurement was obtained and the average of the last 2 readings was used. Fasting vein blood glucose test was performed by a rapid glucose analyzer (BeneCheck PD-G001-2; General Life Biotechnology Co., Ltd., Taiwan, China) and total cholesterol (TC), triglyceride (TG), HDL-C and LDL-C is performed by a rapid lipid analyzer (CardioChek PA Analyzer; Polymer Technology Systems, Indiana, USA). LDL-C is calculated using the values of TC, HDL-C and TG levels in the Friedewald equation.

### Physical activity and sedentary behavior measurement

Physical activity level and sedentary behavior were assessed using questionnaires. The questions on physical activity and sedentary leisure time were adapted from the China Kadoorie Biobank (CKB) [17], and CKB study were adapted from validated questionnaires used in previous other studies [18, 19]. Participants were asked about their usual type and duration of activities in each of the four domains (occupational, commuting, domestic, and leisure-time) in the past 12 months. Occupational related physical activities included farmers (including manual work in the farming season, semi-mechanized work in the farming season, fully mechanized work in the farming season, work outside the farming season) and nonfarmers (including heavy manual work, manual work, standing work, sedentary work). Commuting related physical activities included walking, bicycle, motorbike, private or public transportation (such as bus, car, underground, and ferry). Domestic related physical activities included household activities. Leisure-time related physical activities included Tai-Chi/qigong/leisure walking, jogging/aerobic exercise, ball games, brisk walking/gymnastics/folk dancing, swimming, other exercise (e.g. mountain hiking, home exercise and rope jumping). Every specific physical activity was assigned a specific metabolic equivalent of task (MET) derived from the 2011 Compendium of Physical Activity [20]. For every physical activity level, the amount per day was calculated by multiplying the time spent on the activity in hours per day by the MET of the activity, expressed in MET-h/d. The total amount of physical activity level per day was the sum of all activities.

Participants were asked how many hours per day they spent on sedentary activities during leisure-time such as watching television, reading, playing card games and weaving was defined as sedentary leisure time.

### Carotid ultrasound examination

All carotid ultrasound examinations were obtained by registered ultrasound physicians (certificates issued by Jiangsu Commission of Health) who have worked for at least 5 years. Ultrasound assessment technique followed the recommended guidelines for ultrasound measurement approved by National Center for Cardiovascular Disease. CIMT (mm) were measured for both the right and left common carotid arteries using ultrasound scanner equipped with a variable frequency (5-12 MHz) linear array transducer. Bilateral carotid arteries were scanned with the common carotid artery bifurcation and the near and far walls of the distal 1-1.5 cm proximal to its bifurcation. The IMT of posterior wall with the minimum diameter of carotid artery in diastolic period was used. CIMT values were calculated as the average of three areas on both the right and left carotid artery. Abnormal carotid artery was defined as CIMT ≥1.0 mm at any measurement position [21], and CP was defined as appear one or more of the following conditions in the left or right common carotid artery segment: a thickness of 1.5 mm from the intima-lumen interface to the media-adventitia interface, or more encroaching into the lumen or at least 0.5 mm or 50% compared with the surrounding CIMT values [22].

### Statistical analysis

Characteristics were presented as means (standard deviation) for continuous variables and proportions for categorical variables. The differences characteristics between abnormal and normal carotid artery were compared using one-way ANOVA tests (and using Kruskal-Wallis test when variables were abnormal distribution) for continuous variables or chi-square tests for categorical variables, accordingly.

We used restricted cubic splines [23] with five knots at the 5th, 35th, 50th, 65th, and 95th centiles to flexibly model the association of physical activity level and sedentary leisure time with CIMT. Physical activity level was normalized by Z-score. Multivariable linear regression models were also performed to determine the association of physical activity level (per SD increase) and sedentary leisure time (per SD increase) with CIMT. Multivariable logistic regression models were performed to determine the association of sedentary leisure time (per SD increase), physical activity level (per SD increase), sedentary leisure time quartiles (<1.0, 1.0-<1.7, 1.7-<2.5, and ≥2.5 h/d) and physical activity level quartiles (<5.6, 5.6-<12.2, 12.2-<23.3, and ≥23.3 MET-h/d) with abnormal carotid artery and CP. Multivariable linear regression models were adjusted for, if not sedentary leisure time by, sex, age, BMI, WC, SBP and DBP, current smoker, alcohol drinker, education, household income, fasting glucose, TC, HDL-C, TG and LDL-C and sedentary leisure time. Multivariable logistic regression models were adjusted for, if not physical activity level or sedentary leisure time by, sex, age, BMI, WC, SBP, DBP, current smoker, alcohol drinker, education, household income, fasting glucose, TC, HDL-C, TG, LDL-C, physical activity level and sedentary leisure time.

In additional exploratory analyses, possible modifications of the association of physical activity level (per SD increase) with abnormal carotid artery and CP were also assessed for the variables, including sex (males vs. females), age (<60 vs. ≥60 years), BMI (<24.0 vs. ≥24.0 kg/m^2^), current smoker (no vs. yes), alcohol drinker (no vs. yes), fasting glucose (<7.0 vs. ≥7.0 mmol/L), TC (<6.2 vs. ≥6.2 mmol/L), HDL-C (<1.0 vs. ≥1.0 mmol/L), TG (<2.3 vs. ≥2.3 mmol/L) and LDL-C (<4.1 vs. ≥4.1 mmol/L) by including interaction terms (physical activity plus one of modifier variables) into the logistic regression models.

R software, version 3.5.3 (http://www.R-project.org/) was used for all statistical analyses. A two-tailed *P*<0.05 was considered to be statistically significant in all analyses.

## Patient and public involvement

No patients were involved in the development of the research question, outcome measures, design, recruitment and conduct of this study.

## Results

### Characteristics of the study participants

As shown in Fig. 1, a total of 10,920 participants with an average age of 59.3 years (SD, 8.9 years) were enrolled in the final analysis. The participant characteristics by carotid artery categories were presented in Table 1. The mean physical activity level was 16.8 MET-h/d (SD, 13.8 MET-h/d). Abnormal carotid artery was detected in 5726 (52.4%) participants. Participants with abnormal carotid artery were more likely to be male, the elderly, current smokers, alcohol drinkers, and have higher WC, SBP, fasting glucose, TC, LDL-C, CIMT, and lower DBP, physical activity level, household income level, as compared to those with normal carotid artery.

**Table 1:** Characteristics of the study participants^a^

Variables	Total	Carotid artery		*P* value
		**Normal**	**Abnormal**	
Number (%)	10,920	5194 (47.6)	5726 (52.4)	
Male, No. (%)	4613 (42.2)	1955 (37.6)	2658 (46.4)	<0.001
Age, mean (SD), years	59.3 (8.9)	56.1 (8.8)	62.2 (7.9)	<0.001
WC, mean (SD), cm	86.8 (9.5)	86.3 (9.4)	87.2 (9.6)	<0.001
BMI, mean (SD), kg/m^2^	26.4 (3.4)	26.4 (3.4)	26.4 (3.4)	0.650
SBP, mean (SD), mmHg	163.9 (19.9)	164.1 (20.2)	166.2 (19.3)	<0.001
DBP, mean (SD), mmHg	90.9 (12.2)	91.7 (12.2)	90.2 (12.2)	<0.001
Fasting glucose, mean (SD), mmol/L	6.6 (2.0)	6.4 (1.8)	6.7 (2.1)	<0.001
TC, mean (SD), mmol/L	4.92 (1.18)	4.85 (1.17)	4.98 (1.18)	<0.001
HDL-C, mean (SD), mmol/L	1.34 (0.44)	1.33 (0.45)	1.35 (0.44)	0.073
TG, mean (SD), mmol/L	1.71 (0.97)	1.73 (1.00)	1.69 (0.95)	0.287
LDL-C, mean (SD), mmol/L	2.78 (0.97)	2.71 (0.95)	2.84 (0.99)	<0.001
Household income, No. (%), Yuan/year				<0.001
<10,000	1860 (17.0)	703 (13.5)	1157 (20.2)	
10000-50000	5965 (54.6)	2857 (55.0)	3108 (54.3)	
≥50,000	3095 (28.4)	1634 (31.5)	1461 (25.5)	
Education, No. (%)				<0.001
Primary or lower	6152 (56.3)	2655 (51.1)	3497 (61.1)	
Secondary school	4358 (39.9)	2287 (44.0)	2071 (36.2)	
College or above	410 (3.8)	252 (4.9)	158 (2.7)	
Current smoker, No. (%)	2422 (22.2)	980 (18.9)	1442 (25.2)	<0.001
Alcohol drinker, No. (%)	3214 (29.4)	1401 (27.0)	1813 (31.7)	<0.001
Physical activity level, mean (SD), MET-h/d	16.8 (13.8)	18.6 (14.4)	15.1 (13.1)	<0.001
Sedentary leisure time, mean (SD), h/d	1.9 (1.2)	1.9 (1.2)	1.9 (1.2)	0.562
CIMT, mean (SD), mm	0.7 (0.3)	0.6 (0.1)	0.8 (0.3)	<0.001

^a^WC, waist circumference; BMI, body mass index; SBP, systolic blood pressure; DBP, diastolic blood pressure; TC, total cholesterol; HDL-C, high-density lipoprotein cholesterol; TG, triglyceride; LDL-C, low-density lipoprotein cholesterol; CIMT, carotid intima-media thickness.

### Association of physical activity level and sedentary leisure time with CIMT

**Fig. 2:**
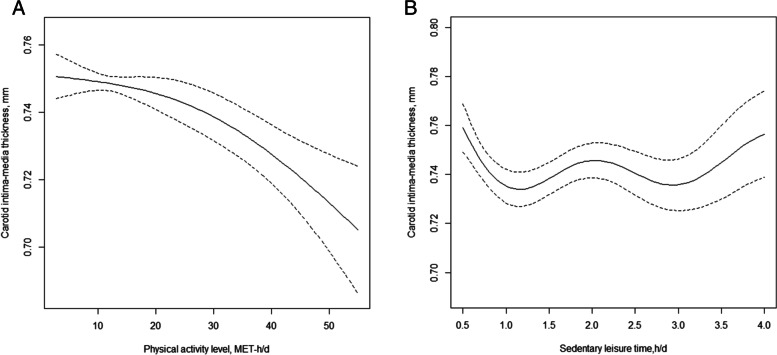
The association of physical activity level (**A**) and sedentary leisure time (**B**) with CIMT^a^. ^a^β of physical activity level (per SD increase:β=-0.0103; 95CI: -0.0154, -0.0053) and sedentary leisure time (per SD increase:β=-0.0004; 95CI: -0.0052, 0.0043) with CIMT was estimated by linear regression, adjusted for, if not sedentary leisure time by, sex, age, BMI, WC, SBP, DBP, current smoker, alcohol drinker, education, household income, fasting glucose, TC, HDL-C, TG, LDL-C and sedentary leisure time. The dotted lines represent the 95% confidence intervals for the spline model. The range of physical activity level was restricted to 1.3 to 55.7 MET-h/d because predictions less than 1.3 MET-h/d (1th percentile) and greater than 55.7 MET-h/d (99th percentile) are based on too few data points. The range of sedentary leisure time was restricted to 0.43 to 4.0 h/d because predictions less than 0.43 h/d (5th percentile) and greater than 4.0 h/d (95th percentile) are based on too few data points

The mean CIMT was 0.7 mm (SD, 0.3 mm) (Table 1). Overall, restricted cubic spline graphs revealed a significant inverse association between physical activity level and CIMT (per SD increase: β=-0.0103; 95% CI: -0.0154, -0.0053) (Fig. 2).

However, there was no significant association of sedentary leisure time with CIMT (per SD increase: β=-0.0004; 95% CI: -0.0052, 0.0043) (Fig. 2).

### Association of physical activity level and sedentary leisure time with abnormal carotid artery and CP

The prevalence of abnormal carotid artery and CP was 52.4% and 43.6%, respectively (Table 2).

**Table 2:** The association of physical activity level and sedentary leisure time with abnormal carotid artery and CP

Variables	Abnormal carotid artery			CP		
	**Event (%)**	**OR (95%CI)** ^a^	***P*** **value**	**Event (%)**	**OR (95%CI)** ^a^	***P*** **value**
Physical activity level, MET-h/d						
Per SD increase	5726 (52.4)	0.908 (0.869, 0.948)	<0.001	4014 (43.6)	0.900 (0.857, 0.945)	<0.001
Quartiles						
Q1(<5.6)	1461 (60.2)	1.0		1130 (53.9)	1.0	
Q2(5.6-<12.2)	1708 (56.6)	0.875 (0.775, 0.928)	0.031	1177 (47.3)	0.745 (0.651, 0.851)	<0.001
Q3(12.2-<23.3)	1377 (50.2)	0.858 (0.758, 0.972)	0.016	904 (39.8)	0.729 (0.635, 0.837)	<0.001
Q4(≥23.3)	1180 (43.2)	0.761 (0.669, 0.865)	<0.001	803 (34.1)	0.679 (0.589, 0.783)	<0.001
* P* for trend			<0.001			<0.001
Sedentary leisure time, h/d						
Per SD increase	5726 (52.4)	1.011 (0.970, 1.054)	0.608	4014 (43.6)	1.036 (0.989, 1.086)	0.240
Quartiles						
Q1(<1.0)	1198 (53.7)	1.0		854 (45.3)	1.0	
Q2(1.0-<1.7)	1397 (50.2)	0.932 (0.824, 1.055)	0.265	964 (41.1)	0.915 (0.796, 1.051)	0.208
Q3(1.7-<2.5)	1719 (54.7)	1.110 (0.983, 1.254)	0.093	1181 (45.3)	1.098 (0.958, 1.258)	0.181
Q4(≥2.5)	1412 (51.1)	0.940 (0.830, 1.066)	0.334	1015 (42.9)	0.975 (0.846, 1.122)	0.720
* P* for trend			0.536			0.951

^a^All reference to normal group, adjusted for, if not physical activity level or sedentary leisure time by, sex, age, BMI, WC, SBP, DBP, current smoker, alcohol drinker, education, household income, fasting glucose, TC, HDL-C, TG, LDL-C, physical activity level and sedentary leisure time.

In the multivariate logistic regression models, the physical activity level was inversely correlated with abnormal carotid artery and CP (per SD increase: OR=0.908, 95%CI: 0.869-0.948, *P*<0.001; OR=0.900, 95%CI: 0.857-0.945, *P*<0.001; respectively). Consistently, when physical activity level was assessed as quartiles, compared with participants in quartile 1, a significantly lower risk of abnormal carotid artery and CP were found in those in quartile 2 (OR=0.875, 95%CI: 0.775-0.928, *P*=0.031; OR=0.745, 95%CI: 0.651-0.851, *P*<0.001, respectively), quartile 3 (OR=0.858, 95%CI: 0.758-0.972, *P*=0.016; OR=0.729, 95%CI: 0.635-0.837, *P*<0.001, respectively) and quartile 4 (OR=0.761, 95%CI: 0.669-0.865, *P*<0.001; OR=0.679, 95%CI: 0.589-0.783, *P*<0.001, respectively). Similarly, the risk of abnormal carotid artery and CP decreased through the quartiles of physical activity level (*P*_for trend_<0.001) (Table 2).

However, there was no significant association of sedentary leisure time with abnormal carotid artery and CP when sedentary leisure time was assessed as a continuous variable (per SD increase) and as quartiles (Table 2).

### Stratified analyses by potential effect modifiers

**Fig. 3:**
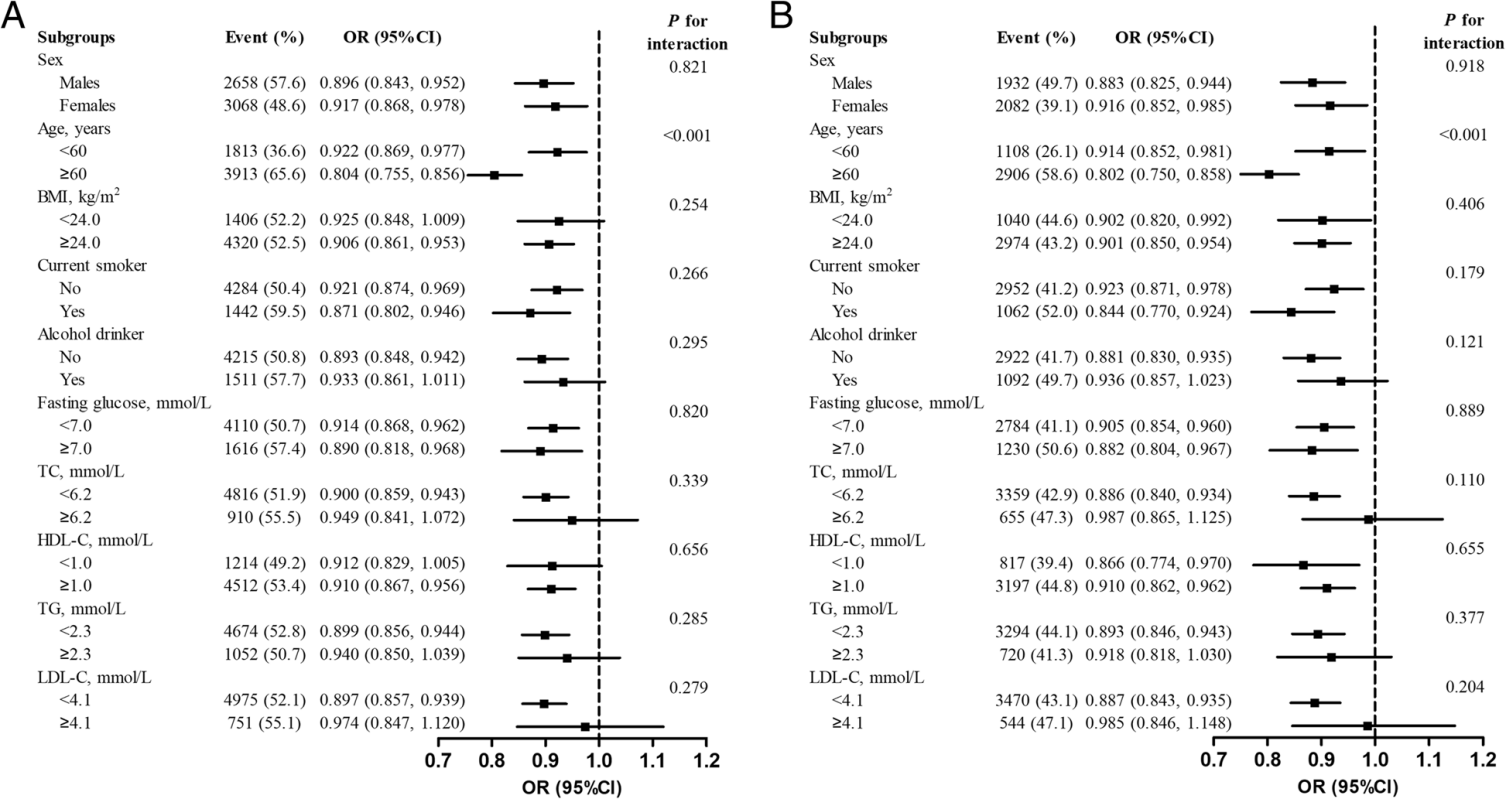
Stratified analyses by potential effect modifiers for the association of physical activity level with abnormal carotid artery (**A**) and CP (**B**)^a^. ^a^Physical activity level was per SD increase; all reference to normal group; adjusted for, if not stratified by, sex, age, BMI, WC, SBP, DBP, current smoker, alcohol drinker, education, household income, fasting glucose, TC, HDL-C, TG, LDL-C and sedentary leisure time

Stratified analyses were performed to assess the association of physical activity level (as a continuous variable) with the risk of abnormal carotid artery and CP in various subgroups (Fig. 3). A significant inverse association between physical activity level (per SD increase) and abnormal carotid artery was stronger in elder participants (≥60 years: OR, 0.804; 95% CI, 0.755-0.856; versus <60 years: OR, 0.922; 95% CI, 0.869-0.977; *P*_interaction_<0.001). Moreover, a significantly stronger inverse association between physical activity level (per SD increase) and CP was also observed in elder participants (≥60 years: OR, 0.802; 95% CI, 0.750-0.858; versus <60 years: OR, 0.914; 95% CI, 0.852-0.981; *P*_interaction_<0.001).

The significantly inverse association of physical activity level with abnormal carotid artery and CP was observed in participants with lower TC (<6.2 mmol/L) or LDL-C (<4.1 mmol/L), while the relationships were attenuated or even diminished among groups with higher TC (≥6.2 mmol/L) or LDL-C (≥4.1 mmol/L), with the test for interaction of TC and LDL-C with physical activity level >0.05 (Fig. 3).

None of the other variables, including sex, BMI, current smoker, alcohol drinker, fasting glucose, TG, HDL-C, showed significant effect modification on the association of physical activity level with the risk of abnormal carotid artery and CP (*P*_interaction_>0.05 for all these stratified variables) (Fig. 3).

## Discussion

The current study demonstrated that physical activity level was inversely associated with CIMT in population with high risk for CVDs. Furthermore, we found that a higher level of physical activity was associated with a lower risk of abnormal carotid artery and CP. A number of previous cohort and cross-sectional studies [13, 24–29] have explored the association between physical activity level and the risk of CAS. Most of them observed a relationship of a higher level of physical activity with less carotid stiffness [24–26], lower CIMT or less progression of CIMT [27, 28]. However, two studies didn’t show the relationship [13, 29]. The inconsistent findings might be due to differences in study design (cross sectional vs. longitudinal); distribution of the target population (children vs. middle-aged adults vs. older adults) [26]. Furthermore, those discrepancies could be attributed to differences in arterial segment [28]; assessment of physical activity, sensitivity and specificity of the ultrasound methods [30]; the extensive use of lipid-lowering medication, antihypertensives, and anticoagulants, which is usually seen in an older adult sample [31]. And the number of study subjects in these previous studies is relatively small [13, 28], therefore, the power for detecting a significant association may be limited, especially in some subgroups. Moreover, previous studies have not fully evaluated any possible effect modifiers in the physical activity-CAS association. Thus, future studies for replication of these findings in randomized controlled trials (RCT) are needed. Our current study including 10,920 participants with high risk for CVDs showed a direct association between physical activity and the carotid artery health and suggested that increased physical activity is important in the prevention of vascular aging, even in population with high risk for CVDs.

There are several established and potential mechanisms which may explain the reduced risk of CAS due to physical activity. First, physical activity reduces insulin sensitivity. Studies have shown that physical activity can enhance insulin sensitivity by regulating the insulin signaling pathway and the effectiveness of fatty acids [32], and decreased insulin sensitivity has been shown to be an independent determinant of CIMT [33]. Second, physical activity improves lipid metabolism or profile of adipokines. It is well known that physical activity promotes weight loss, and body weight was inverse association with CIMT and CP [34], and adipocytokines link excessive body weight and atherosclerosis [35]. Third, physical activity may have direct positive effects on the vasculature structure and function through an increase of nitric oxide (NO) bioavailability to improve endothelial function [36]. Fourth, physical activity reduces blood press. Physical activity have anti-thrombotic/fibrinolytic effects suppression of elevated blood pressure levels [30]. Furthermore, its beneficial effects on systemic inflammation and platelet aggregation were reported [37, 38]. All of these changes affect the structure and function of the carotid artery, suggesting the biological and pathological basis of the association between decreased physical activity and the risk of CAS. Nevertheless, further studies are required to confirm the exact mechanism of physical activity with CAS.

More importantly, we observed that the inverse association between physical activity level and abnormal carotid artery and CP was more stronger in participants with age ≥60. The studies by Boss et al. [25] consistently reported that the association of physical activity with CIMT and stiffness markers appeared to be more pronounced in elder patients (>60 years) than in younger. Previous studies have shown that increased CIMT and CP were more common in the elder population than in the younger [2, 4, 6], and age plays an important role in the progression of CAS. The exact biological mechanism of the interaction between high physical activity and high age is unclear. A reasonable biological explanation was that aging physiology and cardiovascular pathophysiology have common potential pathways of increasing inflammation and insulin resistance [39]. And the downstream effects such as changes in muscles, nerves and cardiovascular system, which will impair exercise capacity and increase susceptibility to cardiovascular disease [40]. Physical activity is a severely underutilized prevention strategy, which can prevent CAS and alleviate some physiological changes that occur with aging [14]. Therefore, our results suggest that elder people with high risk for CVDs should be encouraged to perform more physical activity. Physical activity has been shown to have bebeficial effects on the pathogenesis, symptoms and physical health of patients with dyslipidaemia, and to reduce cholesterol levels [41]. In our current study, we found that the relationship of physical activity with abnormal carotid artery and CP attenuated or even diminished among participants with higher TC or LDL-C. While, the attenuated or even diminished associations among those with higher TC or LDL-C could be due to unmeasured bias or reverse causality which should be further investigated in well-designed population-based prospective studies.

Previous cohort and cross-sectional studies showed inconsistent association of sedentary leisure time with CIMT, abnormal carotid artery and CP. A previous cohort study in 614 healthy men and women reported the proportion of sedentary time was directly associated with baseline common carotid artery (CCA) IMT [28]; Parsons et al. [10] found that sedentary time was positively associated with CIMT, while association with CP was not observed; García-Hermoso et al. [12] reported that sedentary time was positively associated with CIMT and sedentary time in bouts ≥10 min was associated with abnormal carotid artery. In addition, Lazaros et al. and Diaz et al. consistently found that sedentary leisure time (TV viewing time) was independently associated with increased CIMT [11, 42] and the prevalence of carotid plaque [42]. While Kronenberg et al. [14] reported null association. Our study found no significant association of sedentary leisure time with increased CIMT and the prevalence of abnormal carotid artery and CP. Both Kronenberg et al. [14] and our study excluded participants with history of CVDs, which may affect the evaluation of atherosclerosis risk factors by narrowing the variance. On the other hand, such excluded participants may be mostly undergoing interventions with diet and medical drugs which affect the atherosclerosis risk factors in different ways and which would have markedly confounded the analysis. More importantly, previous studies have pointed out that even if the individual’s physical activity level reaches the recommendations long-term sedentary behavior can still increase the risk of chronic diseases [43]. Therefore, the harm to the body of sedentary behavior should not be ignore while strengthening physical activities.

Our study had some potential limitations. First, the study is a cross-sectional design, making it difficult to draw a causal conclusion. Replication of these findings in RCT and additional researches is needed. Second, an additional limitation of the study is the sole use of self-reported physical activity collected using a questionnaire, rather than by a validated physical activity questionnaire or objectively measured physical activity by gold standard approach, such as accelerometer. Third, the excluded participants were older than the enrolled ones, which may underestimate the benefit of physical activity on CAS (Supplemental Table 1). Fourth, due to lack of collecting data of occupational sedentary behavior, one of the explanatory variables, the dose-response relationship between occupational sedentary time and CAS was not able to be assessed. Fifth, self-reported use of lipid-lowering medications is small (0.96%) in our study, therefore we did not include it as a confounding factor in our analysis. Even if they were included, it had no effect on the analysis results of our study. Furthermore, although many covariates were included in the analyses, residual confounding effects from unmeasured measured factors (e.g. diet and medications) cannot be excluded, especially for cross-sectional design. Thus, our study was just hypothesis-generating and more studies are further needed to verify our results.

## Conclusions

In population with high risk for CVDs, physical activity was inversely associated with CIMT, abnormal carotid artery and CP, particularly among the elders. Sedentary leisure time was not associated with CIMT, abnormal carotid artery and CP. These results suggested that physical activity is important for carotid vascular health, and perhaps especially in elder population.

## Supplementary Information

**Additional file 1.**

## Data Availability

The data that support the findings of this study are available from National Center for Cardiovascular Diseases but restrictions apply to the availability of these data, which were used under license for the current study, and so are not publicly available. Data are however available from the authors upon reasonable request and with permission of National Center for Cardiovascular Diseases.

## Abbreviations

CVDs: cardiovascular diseases
PEACE: Patient-Centered Evaluative Assessment of Cardiac Events
CIMT: carotid intima-media thickness
CP: carotid plaque
OR: odds ratio; CI: confidence interval
CAS: carotid atherosclerosis
NCCD: National Center for Cardiovascular Diseases
MI: myocardial infarction
PCI: percutaneous coronary intervention
CABG: coronary artery bypass graft
SBP: systolic blood pressure
DBP: diastolic blood pressure
LDL-C: low-density lipoprotein cholesterol
HDL-C: high-density lipoprotein cholesterol
WC: waist circumference
BMI: Body mass index
TC: total cholesterolx
TG: triglyceride
CKB: China Kadoorie Biobank
MET: metabolic equivalent of task
RCT: randomized controlled trials
NO: nitric oxide
CCA: common carotid artery
