# Supplementary material for: Physical activity and carotid atherosclerosis risk reduction in population with high risk for cardiovascular diseases: a cross-sectional study
Source: BMC Public Health. 2022 Feb 7;22:250. doi: 10.1186/s12889-022-12582-6 (PMC8822633; doi:10.1186/s12889-022-12582-6)
Supplement: Supplementary file 1 — Additional file 1. [file 12889_2022_12582_MOESM1_ESM.docx]

**Supplemental Table 1.** Characteristics of the study participants excluded and enrolled in the current study^a^.

| **Variables** | **Total** | **Participants** | | ***P* value** |
| --- | --- | --- | --- | --- |
|  |  | **Excluded** | **Enrolled** |  |
| Number (%) | 18520 | 7600 (41.0) | 10920 (59.0) |  |
| Male, No. (%) | 7773 (42.0) | 3160 (41.6) | 4613 (42.2) | 0.369 |
| Age, mean (SD), years | 60.1 (8.8) | 61.3 (8.6) | 59.3 (8.9) | <0.001 |
| WC, mean (SD), cm | 86.9 (9.6) | 87.1 (9.7) | 86.8 (9.5) | 0.014 |
| BMI, mean (SD), kg/m^2^ | 26.3 (3.5) | 26.2 (3.5) | 26.4 (3.4) | <0.001 |
| SBP, mean (SD), mmHg | 163.5 (21.0) | 162.9 (22.4) | 163.9 (19.9) | 0.002 |
| DBP, mean (SD), mmHg | 90.5 (12.5) | 89.8 (12.8) | 90.9 (12.2) | <0.001 |
| Fasting glucose, mean (SD), mmol/L | 6.5 (2.0) | 6.5 (2.1) | 6.6 (2.0) | <0.001 |
| TC, mean (SD), mmol/L | 4.90 (1.16) | 4.88 (1.13) | 4.92 (1.18) | 0.035 |
| HDL-C, mean (SD), mmol/L | 1.36 (0.43) | 1.38 (0.41) | 1.34 (0.44) | <0.001 |
| TG, mean (SD), mmol/L | 1.68 (0.96) | 1.63 (0.92) | 1.71 (0.97) | <0.001 |
| LDL-C, mean (SD), mmol/L | 2.77 (0.99) | 2.74 (0.95) | 2.78 (0.97) | 0.022 |
| Household income, No. (%), Yuan/year |  |  |  | <0.001 |
| <10000 | 4244 (22.9) | 2384 (31.4) | 1860 (17.0) |  |
| 10000-50000 | 9929 (53.6) | 3964 (52.1) | 5965 (54.6) |  |
| ≥50000 | 4347 (23.5) | 1252 (16.5) | 3095 (28.4) |  |
| Education, No. (%) |  |  |  | <0.001 |
| Primary or lower | 11500 (62.1) | 5348 (70.4) | 6152 (56.3) |  |
| Secondary school | 6426 (34.7) | 2068 (27.2) | 4358 (39.9) |  |
| College or above | 594 (3.2) | 184 (2.4) | 410 (3.8) |  |
| Current smoker, No. (%) | 4020 (21.7) | 1598 (21.0) | 2422 (22.2) | 0.063 |
| Alcohol drinker, No. (%) | 5384 (29.1) | 2170 (28.5) | 3214 (29.4) | 0.191 |
| ^a^WC, waist circumference; BMI, body mass index; SBP, systolic blood pressure; DBP, diastolic blood pressure; TC, total cholesterol; HDL-C, high-density lipoprotein cholesterol; TG, triglyceride; LDL-C, low-density lipoprotein cholesterol. | | | | |
